# Microplastic Distribution and Transport Mechanisms in the South Sea and East China Sea of Korea

**DOI:** 10.3390/toxics13121070

**Published:** 2025-12-12

**Authors:** Byeongkyu Min, Huiho Jeong, Chon-Rae Cho, Hyeon-Seo Cho

**Affiliations:** 1 Wando Regional Office, National Fishery Products Quality Management Service, 162, Haebyeongongwon-ro, Wando-eup, Wando-gun 59116, Jeollanam-do, Republic of Korea; mbk8760@naver.com; 2 College of Fisheries and Ocean Science, Chonnam National University, 50, Daehak-ro, Yeosu-si 59626, Jeollanam-do, Republic of Korea; 3 Faculty of Environmental & Symbiotic Sciences, Prefectural University of Kumamoto, Tsukide 3-1-100, Higashi-Ku, Kumamoto 862-8502, Japan; huiho.jeong@health.ny.gov; 4 Best Environmental Technology Co., Ltd., 8, Ansan 2-gil, Yeosu-si 59661, Jeollanam-do, Republic of Korea; choccr@bestenviron.co.kr

**Keywords:** microplastic pollution, polymer composition, ocean currents, sediment contamination, East China Sea

## Abstract

Microplastic distribution off the coast of Korea was investigated by collecting and analyzing surface seawater and sediment samples from the South Sea and East China Sea during the summer. Microplastic abundance was found to be highest in the YE area, followed by the EC area and the SS area in both seawater and sediment matrices. The dominant microplastic shapes and sizes were fragments and small particles (0.02–0.3 mm), respectively. This distribution pattern is explained by the transport of low-density, small-sized microplastics from other seas via the high salinity Taiwan Warm Current and Tsushima Warm Current flowing northward from the southern waters of the study area. In contrast, microplastics originating from the Korean landmass along the southern coast were less abundant, likely due to their dispersal by the strong currents of the Jeju Warm Current, Taiwan Warm Current, and Tsushima Warm Current, which carry microplastics toward the Korean Strait. This study highlights the critical role of prevailing ocean currents in shaping the spatial distribution of microplastics, providing insight into sources and transport mechanisms relevant for regional marine pollution management in the Korean coastal waters.

## 1. Introduction

By 2018, global plastic production had reached an alarming 360 million tons, with China alone accounting for 25% of this total. Europe and the United States contributed 17% and 10%, respectively [1]. While a portion of plastic production is reclaimed or reused, it is estimated that approximately 8 million tons of plastic enter the ocean each year [2]. Projections indicate that by 2025, 250 million tons of plastic will be discharged into the ocean [3]. Plastic introduced into the ocean degrades through various processes, primarily photochemical degradation, into different size classes: megaplastics (over 1 m in size), macroplastics (greater than 2.5 cm and smaller than 1 m), mesoplastics (greater than 5 mm and smaller than 2.5 cm), and microplastics (smaller than 5 mm) [4,5]. Among these, microplastics reduced to sizes between 0.02 and 5.0 mm are called secondary microplastics, and those intentionally manufactured in sizes of 0.2 to 5 mm are classified as primary microplastics [6,7,8,9].

Microplastic concentrations in marine environments vary significantly depending on the region, depth, and sampling methods, generally ranging from 0 particles/L to several hundred particles/L. For example, studies have reported concentrations of 221.3 particles/L in bays [10], 4.064 particles/L in coastal waters [11], and 2.43 particles/L in the open ocean [12]. Plastics introduced into the ocean become small, low-density, and have large surface areas, making them highly adsorbent. As a result, they can be transported through the food chain and via atmospheric currents, facilitating their widespread distribution [13,14]. They can also be carried long distances by large ocean currents [15]. Therefore, microplastics that persist in the ocean for extended periods can have a significant impact on marine ecosystems. Microplastics are often difficult to distinguish from organic detritus by size, and some serve as substrates for algae attachment [16]. They are also mistakenly ingested by zooplankton and fry [17]. The ingested microplastics are indigestible and accumulate in organisms, negatively affecting the physiological functions of plankton and potentially threatening their survival [18]. Ultimately, microplastics are transmitted to fish and marine mammals, which are top predators, and potentially to humans, who sit at the top of the food chain. This may have significant implications for human health [19,20].

Asian countries with high levels of plastic use and production, particularly China and Japan, are major contributors to global microplastic pollution [21,22]. Lebreton et al. (2017) [23] reported that 67% of the top 20 rivers responsible for severe plastic outflows are located in Asia, with six of these being located in China (Yangtze River, Xi River, Huangpu River, Dong River, Zhujiang River, Hanjiang River). Furthermore, plastic outflows through rivers in Asia account for approximately 86% of global river plastic discharge. This phenomenon is attributed to the high population density surrounding Asian river basins, high plastic production rates, and high precipitation, all of which contribute to substantial plastic leakage into rivers [24].

The East China Sea, which borders several countries, including China, Japan, and South Korea, is likely the primary recipient of plastics from Asia, where plastic production and use are high. The region is influenced by the China Coastal Current (CCC), the Yellow Sea Cold Water (YSCW), and the strong Yangtze River Discharge Flow (YDF) during the summer. Additionally, warm currents such as the Tsushima Warm Current (TWC) and the Taiwan Warm Current (TC), which flow northward from Southeast Asia, converge in the East China Sea [25,26]. Consequently, plastics introduced from land in various countries are expected to flow into the East China Sea following the characteristics of these currents [27].

Currents flowing into the East China Sea eventually pass through the Korea Strait into the East Sea of Korea [25,26]. Iwasaki et al. (2017) [28] estimated that plastic transport through these currents takes between 122 and 182 days. Since this travel time exceeds the period needed for plastics to break into microplastic-sized fragments, microplastic pollution off the coast of Japan is largely attributed to foreign sources. However, few studies have characterized the occurrence and distribution of microplastics in the upper waters of the Korea Strait, highlighting the need for further studies [28,29].

Understanding the distribution of microplastics in the East China Sea is crucial, not only for the region but also for broader studies across Asia. However, research on microplastic distribution in this area remains insufficient. Therefore, this study sought to examine the distribution characteristics of microplastics (including abundance, polymer types, size, and shape) in the East China Sea. To this end, surface seawater and sediment samples collected from the East China Sea during the summer were analyzed using Fourier transform infrared spectroscopy (FTIR) with array detectors. This study aims to provide a better understanding of how microplastics are introduced into the East China Sea through various ocean currents. The outcomes of this study offer insights into how plastics from the landmasses of different countries are distributed throughout the East China Sea via these currents and are expected to serve as a basis for future studies. Furthermore, compared to the study by Min et al., 2023 [27], which focused on surface seawater microplastics in a limited regional area, this study aims to enhance understanding by encompassing microplastic distributions in both surface seawater and sediments across a broader geographic region including Korea’s South Sea and the East China Sea, utilizing findings from Min et al., 2024 [30]. Additionally, this study integrates analyses of polymer types, shapes, sizes, and the influence of ocean currents to provide a more detailed and comprehensive insight into microplastic pollution dynamics in the study region.

## 2. Materials and Methods

### 2.1. Sampling Method

The study areas selected for investigating the distribution characteristics of microplastics are the southern coast of Korea and the East China Sea, located southwest of Jeju Island. Our study covers a vast expanse of sea, stretching 345.33 km between the eastern and western sites, and 302.41 km between the northern and southern sites. This broad area highlights the extensive scope of our research and the widespread distribution of microplastics that we aim to explore. Given the large size of the study area, securing samples simultaneously posed a significant challenge. To address this, the study was conducted over two separate surveys to ensure a more accurate representation of microplastic distribution.

**Figure 1:**
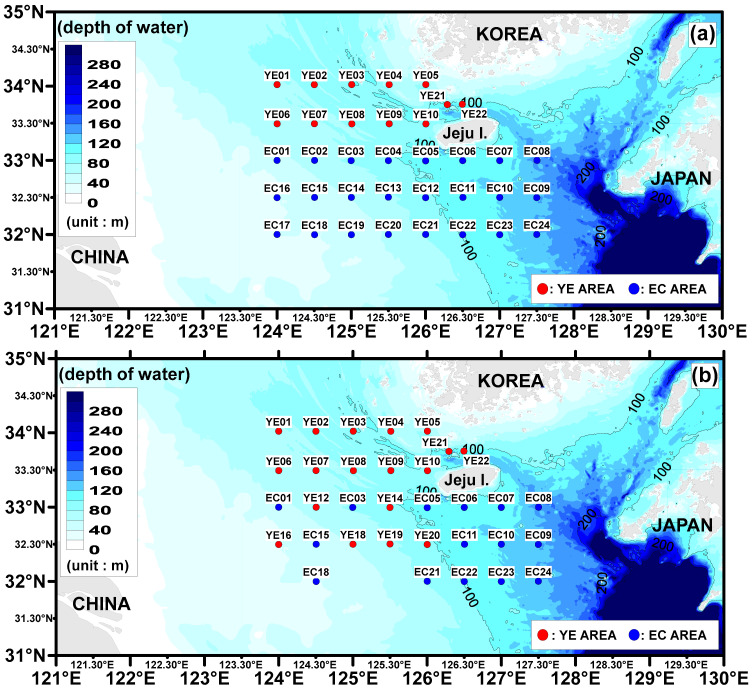
Study areas and sampling stations. (**a**) Surface seawater site. (**b**) Surface sediment site.

The first survey was conducted in the Yellow East Sea area (YE) from 24–27 June 2022 (seawater: 12 sites, sediment: 17 sites). The second survey took place in the East Sea area (EC) from 17–20 August 2022 (seawater: 24 sites, sediment: 15 sites) (Figure 1). The surveys were conducted using the Sae Dongbaek (2996 tons), a training vessel from Chonnam National University. At each site, water mass data were collected through a temperature-salinity (T-S) diagram by measuring the temperature and salinity at all water layers using a CTD (SBE 19, Sea-bird Electronic, Bellevue, Washington, DC, USA).

For seawater sample collection, 100 L of surface seawater from the top 0–20 cm was gathered using a stainless steel bucket with a 20 cm depth. The seawater brought aboard was immediately filtered through a 20 μm stainless steel sieve. Filtered samples were then transferred to the laboratory in 1 L brown glass bottles.

Sediment samples were collected using grabs. A stainless steel spoon was used to collect approximately 500 g (wet weight) of sediment from the 0–3 cm surface layer. The samples were then transferred to the laboratory in brown glass bottles. Containers for storing surface seawater and seabed sediment samples were rinsed with ultra-pure distilled water, which was filtered through a 20 μm stainless steel sieve. The collected samples were stored at 4 °C until further analysis.

### 2.2. Microplastic Pretreatment Method

Surface seawater samples were analyzed according to the “Investigation Guidelines for Qualitative and Quantitative Analysis of Microplastics Remaining in Seawater and Fisheries Life” [31] from the National Institute of Fisheries Science. Sediment samples were examined as described in the “Investigation Guidelines for Qualitative and Quantitative Analysis of Microplastics Residuals in Sediment Deposits” [32]. Additionally, all reagents employed were of analytic grade and contaminant-free. All glassware used in the analysis was washed with ultra-pure distilled water, which was filtered through a 20 μm stainless steel sieve.

#### 2.2.1. Surface Seawater Microplastic Pretreatment Method

Following the collection, materials exceeding 5 mm in size were separated using a sieve of the same mesh size. To remove residual salinity, the samples were gently washed with distilled water and passed through a 20 μm mesh. The rinsed material was transferred into a 500 mL glass beaker and dried for 24 h at 90 °C in a natural convection oven (LDO-080N, Daihan Labtech Co., Ltd., Namyangju, Republic of Korea).

For oxidative digestion, 20 mL of 35% hydrogen peroxide (CAS No. 7722-84-1, JUNSEI Chemical Co., Ltd., Tokyo, Japan), 20 mL of freshly prepared iron sulfate solution, and an appropriate volume of sulfuric acid (CAS No. 7664-93-9, JUNSEI Chemical Co., Ltd., Tokyo, Japan), were sequentially added. The iron sulfate solution was obtained by dissolving 7.5 g of iron sulfate (CAS No. 7720-78-7, JUNSEI Chemical Co., Ltd., Tokyo, Japan), in 500 mL of distilled water. After covering the beaker with aluminum foil, the mixture was allowed to stand in a fume hood for 5 min and subsequently stirred at 180 rpm and 75 °C for 30 min using an MSH-20D stirrer (Daihan Scientific Co., Ltd., Wonju, Republic of Korea). When organic residues persisted, an additional 20 mL of hydrogen peroxide was introduced, and the digestion cycle was repeated until no visible organic matter remained.

Upon completion of digestion, the suspension was filtered through a 20 μm mesh. Ultrapure water (18.3 MΩ · cm; HIQ1, Human Science Co., Ltd., Hanam, Republic of Korea) was used to wash the beaker and transfer all remaining particles onto the mesh. The recovered particles were subsequently moved into a 250 mL separation funnel, followed by the addition of a 6.7 M NaI solution (density 1.6 g/cm^3^; CAS No. 7681-82-5, JUNSEI Chemical Co., Ltd., Tokyo, Japan). After topping the funnel with 100 mL of the NaI solution, the mixture was left undisturbed for 24 h under aluminum foil. The denser bottom fraction was discarded, while the floating fraction was collected by filtration through a 20 μm metal filter (24 mm diameter). The retained material was dried in a desiccator for 24 h prior to further analysis.

#### 2.2.2. Sediment Microplastic Pretreatment

A 250 g (wet weight) aliquot of sediment was placed into a 500 mL glass bottle, and 300 mL of a sodium iodide (NaI) solution (ρ = 1.6 g · cm^−3^) was added. The bottle was capped and shaken vigorously for 1 min to dislodge particles. After agitation, the suspension was allowed to stand for ~30 min to permit the heavier fraction to settle. The supernatant and any material adhering to the upper inner walls were decanted into a 1 L glass beaker; the bottle walls were rinsed with additional NaI solution to maximize recovery. This shake–settle–decant sequence was performed a total of five times to enrich the flotation (microplastic) fraction. The collected flotate was passed through a 20 μm sieve, transferred to a 500 mL beaker, and oven-dried at 90 °C for 24 h in a natural convection dryer (UDO-080N, LABTECH). Subsequent treatments were carried out following the same protocol described in Section 2.2.1.

### 2.3. Polymer Identification

Polymeric composition was determined by Fourier-transform infrared spectroscopy (FT-IR) using a Nicolet iN10 MX system (Nicolet iN10 MX, ThermoFisher, Waltham, MA, USA). The instrument was operated in imaging mode with an array detector capable of automated analysis of particles ≥10 μm. For imaging, the detector aperture was set to 150 μm (width × height). A background spectrum was collected prior to sample acquisition using the instrument’s “Collect background” routine. Spectra were recorded over 450–5000 cm^−1^ at 4 cm^−1^ resolution. Acquired spectra were matched against the OMNIC spectral library (Thermo Fisher, Madison, WI, USA); identifications with ≥80% similarity were classified as plastics.

Based on the National Institute of Fisheries Science guideline (“Investigation Guidelines for Qualitative and Quantitative Analysis of Microplastics Remaining in Seawater and Fisheries Life” [31]), nine polymer classes were targeted: polyethylene (PE), polystyrene (PS), polypropylene (PP), polyester (PY), acrylic, alkyd, polyethylene terephthalate (PET), polyvinyl chloride (PVC), and nylon.

### 2.4. Shape Classification and Size Measurement of Microplastics

Microplastic particles were categorized into five morphological classes—fragments, fibers, spheres, sheets, and pellets—using FT-IR microscopy, following the criteria in [31]. Particle size was quantified as the Feret (maximum) diameter measured with the ruler tool in the OMNIC software (OMNIC, Thermo Fisher, Madison, WI, USA) of the Nicolet iN10 MX instrument.

### 2.5. Quality Assurance/Quality Control (QA/QC)

To evaluate contamination introduced during sampling, pretreatment, and FT-IR analysis, field blanks were taken at a ratio of one blank per ten sampling locations, yielding two field blanks in total. During shipboard sampling, HPLC-grade (or higher) filtered distilled water (Burdick & Jackson) was poured into a 1 L glass bottle to serve as a procedural blank. In addition, a freshly rinsed metal filter paper was inspected for airborne contamination prior to each FT-IR run. Across the two field blanks, three microplastic particles were detected; no particles were observed on the metal filter blank checks.

### 2.6. Comparison with Other Studies

Prior research on microplastic distributions has predominantly used two sampling approaches. The first, a volume-reduced technique, employs net-based samplers such as manta or bongo nets and therefore targets particles larger than the net mesh (e.g., ~300 μm), typically reporting concentrations in the range of 0–1200 particles·m^−3^ [31,33,34,35,36,37]. The second approach relies on bulk water collection (bucket or pump) and subsequent filtration with finer meshes (e.g., 20 μm), which yields substantially different concentration ranges (up to ~152,668 particles·m^−3^ in some reports) [31,37,38,39]. Because mesh size strongly influences reported abundances, comparisons in this study were restricted to results derived from bulk sampling methods. For sediment microplastic comparisons, only studies using grab samplers in non-beach settings were considered.

## 3. Results

### 3.1. Spatial Distribution of Water Temperature and Salinity in the Surface Water

**Figure 2:**
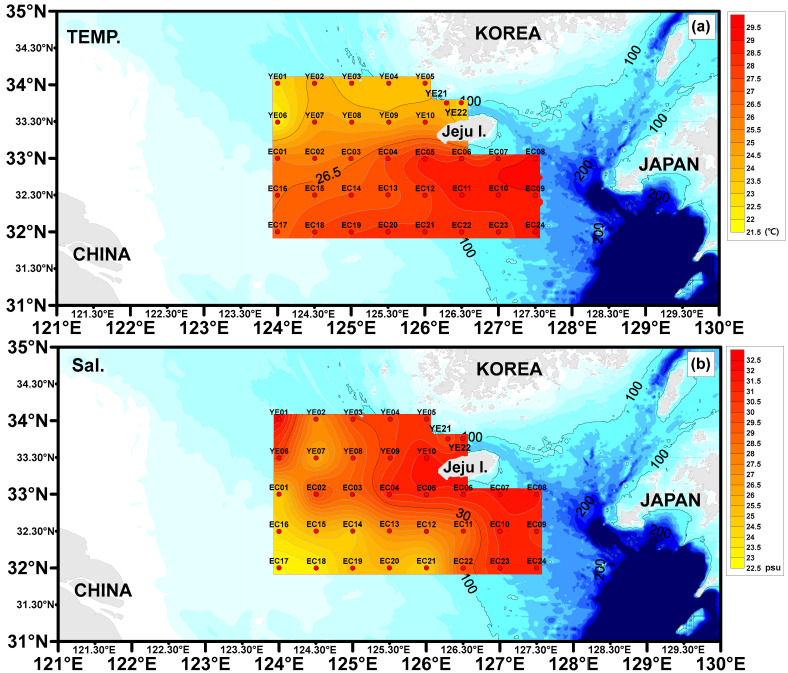
Spatial patterns of surface water temperature (**a**) and salinity (**b**) across the study region.

The recorded water temperatures were as follows: YE area, 21.90–25.42 °C (average 24.04 °C); EC area, 25.79–29.29 °C (average 27.78 °C) (Table 1, Figure 2). The sites with lower water temperatures are likely influenced by the East China Sea Coastal Current, while those with higher temperatures are likely affected by the Kuroshio Current.

The salinity concentrations were as follows: YE area, 24.13–32.33 psu (average 29.39 psu); EC area, 22.96–31.86 psu (average 28.08 psu) (Table 1, Figure 2). Low salinity concentrations observed in the southwestern part of the study area are likely influenced by the Yangtze River Discharge Flow. In contrast, higher salinity concentrations in the southeastern region are believed to result from the influence of the Kuroshio Current.

### 3.2. Microplastic Abundance

**Figure 3:**
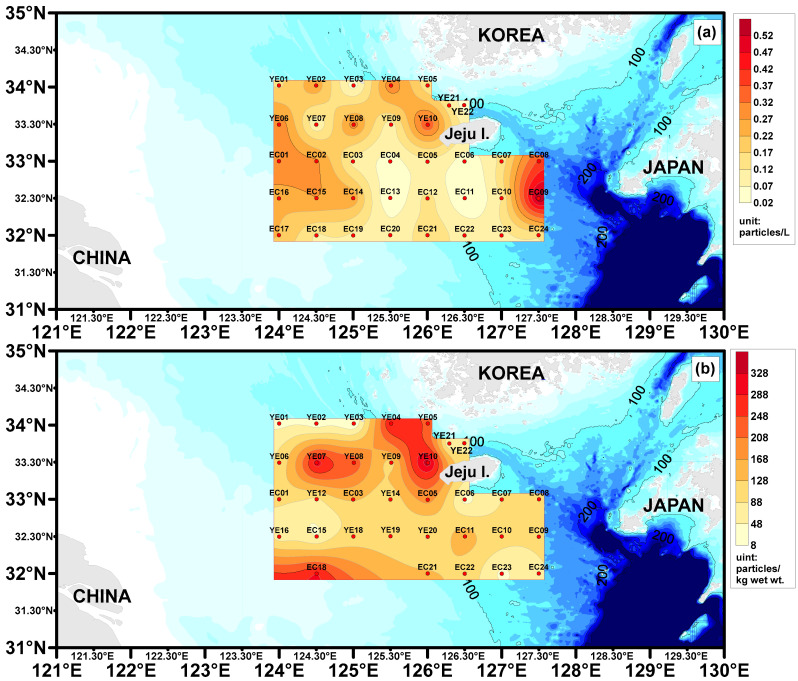
Surface seawater Microplastic abundance (**a**) and Surface sediment Microplastic abundance (**b**) distribution chart for the study areas.

Surface seawater microplastic abundance ranged as follows: YE area, 0.04–0.38 (average 0.18 ± 0.10) particles/L; EC area, 0.02–0.56 (average 0.17 ± 0.13) particles/L (Table 1, Figure 3). Relatively high microplastic abundance was observed at the EC08 (0.56 particles/L) and EC09 (0.38 particles/L) sites, likely influenced by the northward movement of the Tsushima Warm Current into the study area. Additionally, a site near the region impacted by the Yangtze River Discharge Flow exhibited slightly higher microplastic abundance compared to other sites (EC01, EC02, EC14, EC15, EC16, and EC17 sites), a finding that warrants further exploration in the discussion. For surface sediment, microplastic abundance was as follows: YE area, 8.0–356.0 (average 124.4 ± 95.0) particles/kg ww; EC area, 32.0–292.2 (average 114.9 ± 62.6) particles/kg ww. The YE04 (292.0 particles/kg ww), YE05 (236.0 particles/kg ww), and YE10 (356.0 particles/kg ww) sites, located near the Korean coastline, exhibited slightly higher microplastic abundance compared to other sediment sites, presenting a contrasting trend to the surface seawater results.

### 3.3. Microplastic Polymer Types

**Figure 4:**
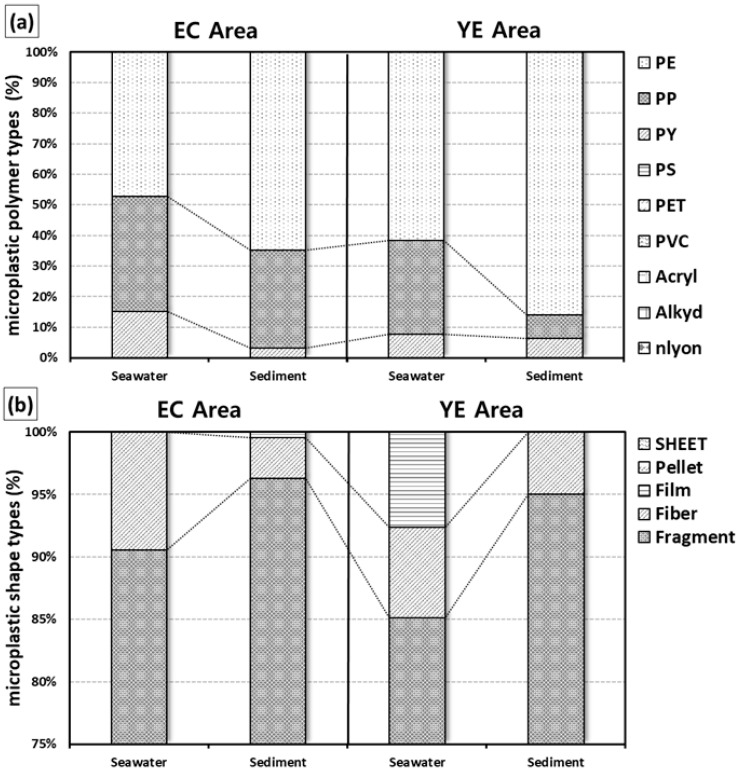
Surface seawater and surface sediment microplastic polymer types (**a**) and microplastic shapes (**b**) in the study areas.

In surface seawater, only three out of nine polymer types were detected across the YE and EC areas: PP (polypropylene), PE (polyethylene), and PY (polyester). Among these, PE was the most prevalent, accounting for 66% and 49% of the total microplastics in the YE and EC areas, respectively. In the EC area, however, PP was slightly more abundant (39%) compared to other regions. For surface sediment, a similar pattern was observed, with PP dominating the detected polymer types. The proportions of PE in the sediment were as follows: YE area, 87%; EC area, 63%. These percentages were notably higher than those observed in the surface seawater samples (Table 1, Figure 4a).

### 3.4. Microplastic Shapes

In surface seawater, the detected microplastic shapes varied slightly across the study areas. In the YE area, only fragments, fibers, and filters were observed, while in the EC and SS areas, only fragments and fibers were detected. Fibers constituted the highest proportion of microplastics in all areas, accounting for 87% in the YE area and 91% in the EC area. For surface sediment, fragments were the dominant microplastic shape in the YE and EC areas, with proportions of 97% and 96%, respectively, similar to the results observed in surface seawater. No clear horizontal distribution patterns were identified for microplastic shapes in either surface seawater or surface sediment (Table 1, Figure 4b).

### 3.5. Microplastic Size

**Figure 5:**
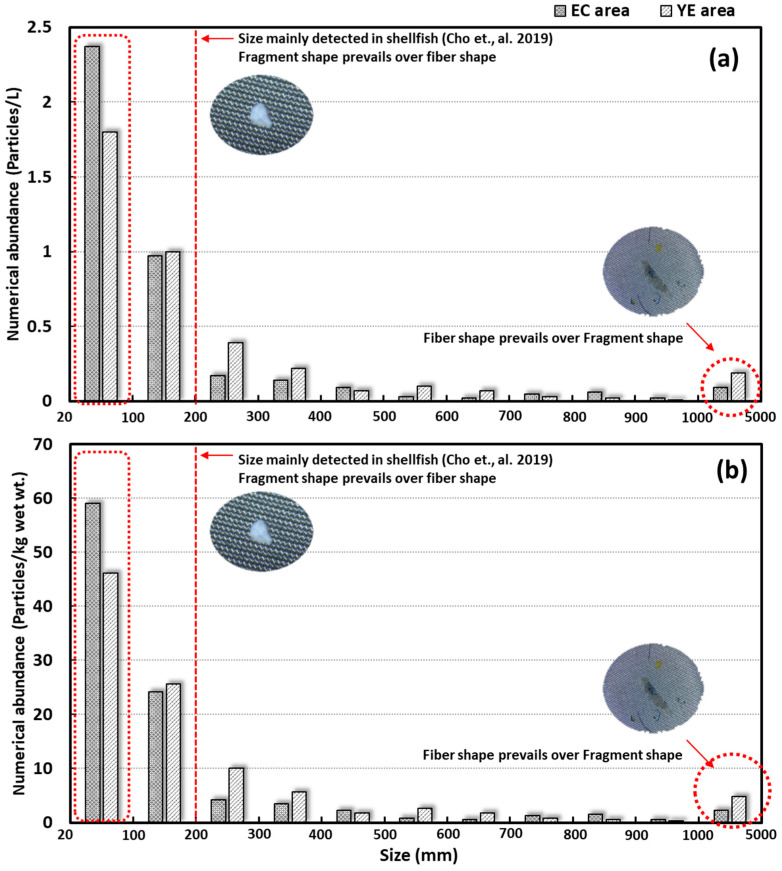
Size distribution profiles of microplastics in surface seawater and surface sediments [38]: (**a**) surface seawater and (**b**) surface sediments.

Microplastics collected from surface seawater were categorized into four size ranges: 0.02–0.3 mm, 0.3–0.6 mm, 0.6–1.0 mm, and 1.0–5.0 mm. The smallest fraction (0.02–0.3 mm), previously identified as a size class prone to trophic transfer in shellfish, was also included [38], was the most abundant. This group accounted for over 85% of microplastics in the YE and EC areas. In surface sediment, all study areas (YE, EC) displayed the same four size groups. Similar to the surface seawater results, the 0.02–0.3 mm size group was dominant, representing over 93% in the YE area and 82% in the EC area (Table 1, Figure 5).

Both surface seawater and surface sediment samples confirmed that fragments were the predominant microplastic type, particularly in the smaller size ranges. Within the largest size group (1.0–5.0 mm), fibers were slightly more abundant than fragments, likely due to their relatively larger surface area. Fibers, originating from sources such as clothing and fishing nets, appeared to contribute more significantly to this size group than fragments.

### 3.6. Comparison with Other Literature

The microplastic abundance observed in this study was evaluated by comparing the results from the East China Sea, the southern coast of Korea, and the southern waters of Jeju Island with those reported in other marine environments. The comparisons were categorized into three areas: Bay, Coastal, and Open Sea, to assess relative levels (Table 2 and Table 3).

In the bay areas, the microplastic levels in this study were significantly lower than those reported in Cheonsu Bay, Hampyeong Bay, Deukryang Bay, Youngil Bay, and Gwangyang Bay in Korea [11], Jiaozhou Bay in China [39], and Tokyo Bay in Japan [10]. The differences ranged from approximately 4 times lower (Cheonsu Bay) to over 1000 times lower (Tokyo Bay). However, the extremely high levels reported for Tokyo Bay may have been overestimated due to the small sample volume (1 L) used in that study, compared to the 100 L average sample volume used in this and other studies. Excluding Tokyo Bay, the differences ranged from 4 times lower (Cheonsu Bay) to 17 times lower (Gwangyang Bay).

In coastal areas, the microplastic levels in this study were higher than those reported for the South China Sea [40] and the southern part of the Taiwan Strait [41], but comparable to those in the northern Taiwan Strait [42]. However, the levels were lower than those observed along the coasts of Ulsan, Incheon, and Busan in Korea [11], the Yellow Sea, South Sea, and East Sea of Korea [43], the Southwest Sea of Korea [27], the China Coast [44], the North Yellow Sea of China [45], and the coastal areas of Malaysia [46]. The differences in microplastic abundance ranged from 25 times lower (China Coast) to similar levels (Malaysia Coast). In the open sea, the microplastic levels in this study were comparable to those reported in the Atlantic Ocean [47] but lower than those in the Greenland Sea [12]. Overall, the surface seawater results in this study demonstrated the following ranking in terms of microplastic abundance: Bay > Coastal > Open Sea.

For surface sediment, the microplastic levels in bay areas were higher than those reported for Qinzhou Bay (mangrove areas) in China [48], Lim Chu Kang Bay in Singapore [49], and Tokyo Bay in Japan [10]. However, they were lower than those observed in Qinzhou Bay (mangrove side) [48], Sanggou Bay [50], Laizhou Bay [51], Belgian Bay [52], the Pearl River Estuary [53], and Gwangyang Bay in Korea [30]. The differences ranged from approximately 3 times lower (Belgian Bay) to 90 times lower (Pearl River Estuary).

In coastal areas, the microplastic levels were comparable to those in the Belgian Coastal Zone [52], the French Atlantic Coastal Zone [54], and the Spanish Mediterranean Coastal Zone [55]. However, they were lower than levels reported for Korea’s West Coast tidal zones [56], the North Yellow Sea of China [57], the Maowei Sea [58], the Taiwan Strait [40], and the North African Coast [59]. The differences ranged from approximately 7 times lower (Taiwan Strait) to 40 times lower (Korea’s West Coast). The microplastic levels in this study were higher than those in the Southern Baltic Sea [60] but lower than those reported for the Western Pacific Ocean [61]. In conclusion, based on the findings of this study, the surface seawater microplastic levels were ranked as follows: Bay > Coastal > Open Sea.

**Table 2:** Comparison of microplastic abundances in surface seawater reported by earlier studies conducted in the South Sea and East China Sea of Korea (unit: particles/L).

Nation	Area		Investigation	n	Collected Vol.	Abundance	Reference
					(Mesh Size)	(Mean/SD)	
China	Jiaozhou	Bay	2017	14	50 L (20 μm)	1.602 ± 1.274	[39]
Korea	Cheonsu		2016/2017	5	100 L (20 μm)	0.784 ± 0.272	[11]
Korea	Hampyeong		2016/2017	5	100 L (20 μm)	1.548 ± 0.211	[11]
Korea	Deukryang		2016/2017	5	100 L (20 μm)	1.146 ± 0.423	[11]
Korea	Youngil		2016/2017	5	100 L (20 μm)	1.688 ± 0.496	[11]
Korea	Gwangyang		2016/2017	5	100 L (20 μm)	2.362 ± 1.022	[11]
Korea	Gwangyang		2020	5	100 L (20 μm)	3.17 ± 1.123	[30]
Japen	Tokyo		2021	4	1 L (20 μm)	221.3 ± 189.5	[10]
Korea	Ulsan	Coastal	2016/2017	5	100 L (20 μm)	1.764 ± 1.006	[11]
Korea	Incheon		2016/2017	5	100 L (20 μm)	4.064 ± 1.075	[11]
Korea	Busan		2016/2017	6	100 L (20 μm)	1.020 ± 0.279	[11]
Korea	Yellow Sea		2018	9	200 L (20 μm)	0.266 ± 0.459	[43]
Korea	South Sea		2018	5			
Korea	East Sea		2018	8	200 L (20 μm)	0.289 ± 0.280	[43]
Korea	Southwest Sea		2020	23	30 L (20 μm)	0.46 ± 0.27	[27]
Korea	SS area		202	15	100 L (20 μm)	0.01 ± 0.09	[30]
China	China		-	16	100 L (50 μm)	4.5 ± 1.8	[12]
China	North Yellow Sea		2016	50	25 L (30 μm)	0.545 ± 0.282	[45]
China	South China Sea		2021	29	200 L (64 μm)	0.103 ± 0.098	[40]
Chinese Taipei	Taiwan Strait (the northern area)		2021	33	1000 L (44 μm)	0.174	[42]
Chinese Taipei	Taiwan Strait (the southern area)		2017	19	100 L (100 μm)	0.035 ± 0.004	[41]
Malaysia	Malaysia		2018	-	2.041 L/s (20 μm)	0.211 ± 0.104	[46]
Atlantic Ocean		Open sea	2014	23	2.6 m^3^ (10 μm)	0.013–0.501	[47]
Greenland	Greenland Sea (GSG)		2018	20	100 L (50 μm)	2.43 ± 0.84	[12]
	Greenland Sea (EGC)					1.19 ± 0.28	
this study	YE area		2022	12	100 L (20 μm)	0.18 ± 0.10	-
	EC area			24		0.17 ± 0.13	

**Table 3:** Comparison of microplastic concentrations in surface sediments with findings from previous studies in the South Sea and East China Sea of Korea. (unit: particles/kg ww).

Nation	Area		Investigation	n	Abundance	Reference
					(Mean/SD)	
China	Qinzhou (mangrove side)	Bay	spoon	7	1298 ± 2207	[48]
China	Qinzhou (mangrove in)		spoon	7	42.9 ± 26.8	[48]
China	Sanggou		Van Veen grab	8	1674 ± 526	[50]
China	Laizhou		Van Veen grab	58	461.6 ± 167	[51]
China	Lim Chu Kang		-	7	36.8 ± 23.6	[49]
China	Belgian		Van Veen grab	11	167	[52]
China	Perl River Estuary		grab	20	4655 ± 1493	[53]
Japen	Tokyo		grab	4	0.016 ± 0.0008	[10]
Korea	Gwangyang		Van Veen grab	5	462.4 ± 143.9	[30]
Korea	West Coast tidal	Coastal	spoon	7	2191	[56]
Korea	SS area		grab	12	50.5 ± 29.7	[30]
China	North Yellow Sea		Box sampler	28	499.8 ± 370.1	[57]
China	Maowei Sea,		-	10	520–2310	[58]
Chinese Taipei	Taiwan Strait		grab	33	16–382	[42]
Belgian	Belgian coast		-	6	97.2	[52]
French	Atlantic coastal		box-core	3	67 ± 76	[54]
The Mediterranean	North African coasts		Corer/Visual	4	182.7–649.3	[59]
	The Spanish Mediterranean		Box-corer	10	113.2 ± 88.9	[55]
Pacific Ocean	The Western Pacific Ocean	Open sea	box corer	15	240	[61]
Baltic Sea	The Southern Baltic Sea		-	-	15 ± 10	[60]
this study	YE area		grab	15	124.4 ± 95.0	-
	EC area		grab	18	114.9 ± 62.6	

## 4. Discussion

**Figure 6:**
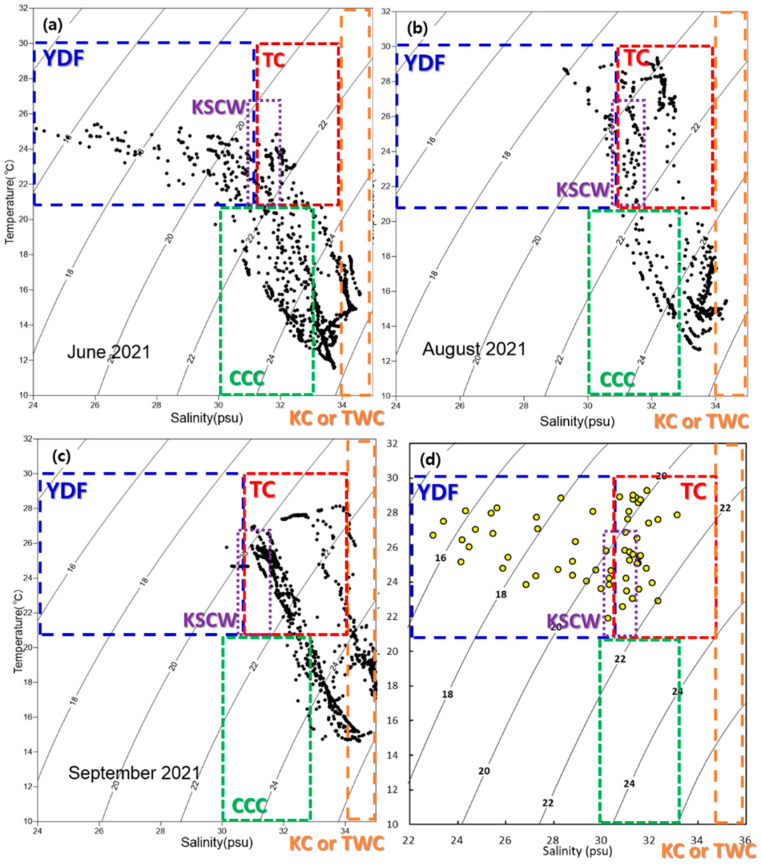
Results of water mass analysis. T-S diagram in the study area: (**a**) seawater column in YE area, (**b**) seawater column in EC area, (**c**) seawater column in SS area, (**d**) surface seawater results for the YE, EC, and SS areas. Yangtze River Discharge Flow (YDF).

Understanding ocean currents in the study area is essential for investigating the distribution characteristics of microplastics. Although microplastics can sometimes settle shortly after entering the ocean, their low density often allows them to be transported by ocean currents [28,62]. Consequently, studies on microplastic distribution must incorporate ocean current data specific to the study region. To explore the distribution characteristics of microplastics, analyses of water masses and ocean currents were conducted. In addition, among the results of Min et al. (2024) [30], those for Korea’s southern coastal waters (SS area) are closest to this study; they are similar in terms of timing, materials, and methods, and the results were discussed. Water mass analysis utilized a T-S diagram, derived from water temperature and salinity measurements at each sampling site. This analysis identified the interactions of several major currents, including the Yangtze River Discharge Flow (YDF), the Taiwan Warm Current (TC), the Kuroshio Current (KC), and the Yellow Sea Cold Water (YSCW) [25,63,64] (Figure 6). Based on these findings, four distinct ocean current groups were determined to influence the study area: the Yangtze River Discharge Flow group (YDF group), the Taiwan Warm Current group (TC group), the Yellow Sea Cold Water group (YSCW group), and the Chinese Coastal Current group (CCC group).

**Figure 7:**
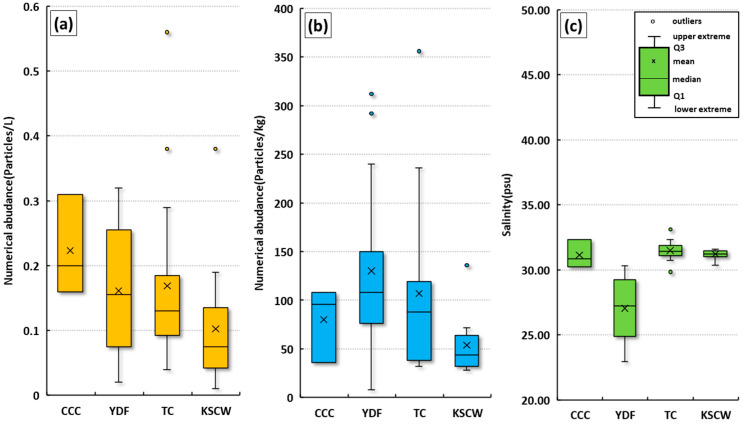
Comparison of surface seawater microplastic abundance (**a**), surface sediment microplastic abundance (**b**), and surface seawater salinity (**c**) by group in the study areas.

The abundance of microplastics in surface seawater and surface sediments was compared by ocean current group (Figure 7). In surface seawater, the abundance followed this order: CCC group (average 0.10 ± 0.08 particles/L) > YDF group (average 0.16 ± 0.16 particles/L) > TC group (average 0.22 ± 0.20 particles/L) > YSCW group (average 0.10 ± 0.08 particles/L). In surface sediments, the abundance followed this order: YDF group (average 130.2 ± 108.0 particles/kg ww) > TC group (average 107.0 ± 88.0 particles/kg ww) > CCC group (average 80.0 ± 96.0 particles/kg ww) > YSCW group (average 53.8 ± 44.0 particles/kg ww). Each ocean current group is expected to influence the abundance of microplastics, and the distribution characteristics of microplastics in each group are discussed in detail.

### 4.1. Taiwan Current Warm Water Group (TC) Group

At the EC08 and EC09 sites, located in the southeastern sea around Jeju Island, the abundance of microplastics in surface seawater was high (Figure 2). However, the abundance in surface sediments appeared to be low. This area is characterized by a faster flow rate, with the current flowing toward the Korea Strait [43]. The faster flow rate suggests that microplastics may remain suspended in the surface layer without sinking to the seabed. The peaks in microplastic abundance are located far from surrounding land (Jeju Island, Korea: about 100 km; Fukue Island, Japan: about 100 km), making evidence of land-based inflow insufficient. Consequently, we considered whether the microplastics could have been introduced by marine activities, such as fishing or other human-induced activities. To monitor fishing activities, an automatic identification system (AIS) was installed at the top of Halla Mountain on Jeju Island. This system tracked the movement of various fishing vessels, including longline, purse seine, gillnet, jigging, trawl, and pot fishing boats, throughout 2019 [65]. In the waters corresponding to the EC08 and EC09 sites, where the surface seawater microplastic abundance was highest, the AIS data showed minimal fishing activity. As a result, we concluded that the microplastics in this area were most likely introduced by ocean currents from other regions, rather than by local marine activities (e.g., fishing). This led us to consider other potential factors influencing the microplastic distribution.

The salt concentrations at the EC08 (31.86 psu) and EC09 (30.73 psu) sites were higher (Figure 2b) compared to those in other groups, resulting in a higher seawater density (1.03 g/cm^3^). The PE and PP microplastics detected in this group constitute over 90% of the total, with densities ranging from 0.85 to 0.98 g/cm^3^, which are relatively lower than seawater. Higher salinity increases the buoyancy of microplastics, causing them to remain suspended on the surface of seawater [44]. This suggests that the microplastics may have been introduced by ocean currents from other regions. Additionally, due to the fast flow rate in this area, the likelihood of floating microplastics sinking to the seabed is lower than in other regions, resulting in a higher abundance in surface seawater. Abidli et al. (2018) [66] reported that microplastics transform into black and transparent colors over time in the marine environment. Martí et al. (2020) [67] also found that microplastics transported to distant seas become smaller fragments, turning white or bright teal due to photooxidation. The results of this study align with these findings, as most of the microplastics in the Taiwan Current Group were found to be white or black, with small-sized microplastics making up a high percentage (79% on average) [27].

Consequently, the TC group is influenced by the Taiwan Warm Current and the Tsushima Warm Current, which move northward from the southwestern waters of Jeju Island. These currents, which have high salinity, increase the buoyancy of microplastics. It is believed that the microplastics were introduced from overseas waters and then transported to the Korea Strait by ocean currents, rather than settling on the seabed due to the fast flow rate in the TC Group waters.

### 4.2. Yangtze River Discharge Flow (YDF) Group

The findings from the July and August discharges of the Datong floodgates, the last floodgates downstream of the Yangtze River in China, show that the annual average discharge was 29,163 m^3^/s in 2019 and 34,763 m^3^/s in 2020 [68]. In July, when the maximum discharge occurred in both years, the discharge reached 69,744 m^3^/s in 2019 and 81,807 m^3^/s in 2020, more than twice the average annual discharge. High microplastic concentrations are found in the Yangtze River surface water system, ranging from 195,000 to 900,000 particles/km^2^ (average 492,000). These microplastics are deposited in the Yangtze River [69] but are eventually released into the ocean through the Yangtze River estuary. The surface layer of the Yangtze River estuary averages 8.55 ± 1.79 particles/L [70]. Studies conducted in waters approximately 120 km from the study area report microplastic concentrations ranging from 0.2 to 0.6 particles/L [71]. In this study site, located approximately 220 km from the Yangtze River estuary, the YDF group averaged 0.16 particles/L, suggesting that microplastic abundance decreases slightly with increasing distance from the estuary.

Microplastics introduced into the Yangtze River estuary are somewhat less buoyant due to the low salinity. Larger microplastics and high-density plastics (e.g., acrylic, polyethylene terephthalate (PET), polyvinyl chloride (PVC), alkyd, nylon) tend to sink to the seabed, while smaller microplastics and low-density plastics (e.g., polypropylene, polyethylene) remain in the surface layer. To confirm this, the mechanism of microplastic sinking to the seabed was examined. Zhang (2017) [72] reported that stratification and mixing at the boundary between river water and seawater are crucial factors affecting the movement of plastic debris. Specifically, the difference in specific gravity between freshwater (1.00 g/cm^3^) and seawater (1.03 g/cm^3^) affects buoyancy, causing plastic debris to deposit in the estuary. Furthermore, low circulation and high sedimentation rates in estuaries contribute to the floating or sinking of plastics.

In the Yangtze River estuary, microplastic abundance was 2378.80 particles/kg [73], significantly higher than the average microplastic abundance of 130 particles/kg ww in the YDF group in this study area. Additionally, Zhang et al. (2019) [74] confirmed that microplastic abundance in sediments decreases as one moves away from the Yangtze River estuary toward the open sea. It is believed that a large volume of microplastics flows into this study area after being released from the Yangtze River into the ocean. However, large-sized and high-density plastics are thought to settle near the mouth of the Yangtze River due to low salinity, while low-density microplastics are transported into this study area through buoyancy. The microplastic polymer type analysis in this study supports this hypothesis, as low-density polypropylene and polyethylene account for more than 90%. The results from the microplastic sediment analysis show that the TC group had slightly higher microplastic abundance than the YDF group, while the surface seawater results indicated a higher abundance in the YDF group than in the TC group. This suggests that the distribution of microplastics is influenced by the buoyancy effects caused by salinity differences in the YDF group.

### 4.3. Korea Southern Coastal Water (KSCW) Group

Lee and Choi (2009) [75] reported that in the summer, Korea’s Southern Coastal waters and the Tsushima Warm Current meet in the waters of the KSCW group, forming a thermohaline circulation. It was confirmed that the SS04, SS05, SS08, and SS09 sites are located in this study area. Although no precise results were obtained from the microplastic abundance analysis in this study, observations of microplastic size and shape suggested a uniform size and shape group at the aforementioned sites, with little diversity. This trend is distinct compared to other sites. Furthermore, Eo et al. (2021) [43] compared sea areas similar to this study area and found higher microplastic abundance in regions (both coastal and offshore) near the thermohaline circulation boundary. In contrast, the thermohaline circulation boundary area in this study showed lower microplastic abundance, aligning with the trends observed here.

Furthermore, Min et al. (2024) [30], in a sub-study of this research, predicted that microplastics entering Gwangyang Bay in Korea would exit through the Yeosu Strait and move into the southern coastal waters of this study area, with some eventually depositing in the sediment. Since most microplastics are carried toward the Korean Strait by the Jeju Warm Current and Tsushima Current, which are particularly strong in summer, the southern coastal waters are considered to have much lower microplastic abundance compared to other areas.

As described above, our findings suggest that the boundary formation in the sea area influenced by thermohaline circulation, along with the effects of the Jeju Warm Current and Tsushima Current, which are especially strong in summer, may result in lower microplastic abundance in this area. This is likely due to the currents moving northward from the southern seas of Jeju Island, rather than an influx from the Korean coastline.

### 4.4. Chinese Coastal Current (CCC) Group

The CCC group includes only two sites, YE01 and YE06, which makes it challenging to assess the microplastic distribution characteristics. However, additional factors were considered when analyzing data from these sites. Both YE01 and YE06 exhibit relatively low water temperatures, which are conducive to the presence of cold-water zooplankton species, such as Calanus sinicus and Centropages abdominalis. These species were identified in concurrent zooplankton studies at these sites (this study’s results) and in previous studies by Wang and Zuo (2003) [76] and Soh and Choi (2004) [77]. This suggests that the Chinese Coastal Current, which brings cooler waters, influences these areas [40]. Consequently, microplastics are more likely to be transported into this study area via the Chinese Coastal Current, which has a higher flow rate, rather than through the Yangtze River Discharge Flow.

Additionally, an experiment conducted for another research purpose examined the presence and characteristics of microplastics in zooplankton that mistake them for food. The results showed that 54 out of 900 zooplankton (18%) contained microplastics in their bodies. While fibers were found at a slightly lower rate than fragments, some fibers were longer than the body length of the zooplankton. Since previous studies have reported that zooplankton in natural environments ingest large plastic pieces [78], further research should focus on understanding the mechanisms by which zooplankton consume fibers longer than their body length.

Moreover, because microplastics were found in 18% of the zooplankton, it is likely that the movement and transport of microplastics by zooplankton will affect the study area, as they carry microplastics along with them while moving with the currents from other regions. However, due to the limited number of sites in the CCC group, determining the characteristics of microplastic distribution at these two sites remains difficult. Therefore, identifying the distribution patterns will be addressed in future research.

## 5. Conclusions

This study investigated microplastic distribution characteristics in surface seawater and sediments of the South Sea and East China Sea off Korea during summer.

Polypropylene (PP) was identified as the dominant polymer, constituting over 47% of microplastics in seawater and more than 63% in sediments. Microplastic abundance was higher in the YE area compared to the EC area for both seawater and sediments. Fragments dominated the microplastic shapes, accounting for the majority in both matrices, while small-sized particles (0.02–0.3 mm) were highly prevalent. The study suggests that larger, denser plastics tend to accumulate near estuaries such as the Yangtze River, whereas smaller, low-density microplastics (e.g., PE, PP, PS) are transported northward by the Taiwan and Tsushima Warm Currents. Land-derived microplastics along Korea’s southern coast appear to be less abundant due to dispersal by strong regional currents toward the Korean Strait. Overall, the findings highlight that small, low-density fragments influenced by multiple oceanic currents predominate and are likely transported from the study area through the Korea Strait into the East Sea, providing important insights for regional marine pollution management and policy development.

This study provides critical insights into the distribution characteristics of microplastics in the South Sea and East China Sea, revealing the impact of key ocean currents on the transport and fate of microplastics in these waters. The data on abundance, polymer composition, shape, and size inform potential sources and ecological risks, which are essential for developing targeted regional pollution control measures. By elucidating the pathways and accumulation patterns of microplastics, the study contributes valuable baseline knowledge that supports effective marine environmental protection and sustainable management policies in the region.

## Figures and Tables

**Table 1:** Summary of results for surface seawater temperature, salinity, and the abundance, polymer composition, size distribution, and morphological categories of microplastics in both surface water and sediments.

	Seawater		Sediment	
	EC Area	YE Area	EC Area	YE Area
Temperature [unit: °C]				
	27.78 ± 1.02 (25.79–29.29)	24.04 ± 0.87 (21.90–25.42)	-	-
Salinity [unit: psu]				
	28.08 ± 3.07 (22.96–31.86)	29.39 ± 2.16 (24.13–32.33)	-	-
Abundance [unit: seawater-particles/L/sediment-particles/kg ww]				
	0.17 ± 0.13	0.19 ± 0.12	114.9 ± 62.6	153.3 ± 120.1
	(0.02–0.56)	(0.04–0.38)	(32.0–292.0)	(8.0–356.0)
Polymer composition * [unit: seawater-%(particles/L)/sediment-%(particles/kg ww)]				
PE (Polyethylene)	49	66	63	84
	(0.08 ± 0.06)	(0.11 ± 0.07)	(74.4 ± 63.2)	(132.3 ± 109.1)
PP (Polypropylene)	39	26	33	11
	(0.06 ± 0.06)	(0.06 ± 0.05)	(36.8 ± 28.9)	(11.3 ± 11.3)
PY (Polyester)	12	8	4	5
	(0.03 ± 0.03)	(0.01 ± 0.02)	(3.7 ± 5.9)	(9.7 ± 14.6)
Shape ** [unit: seawater-%(particles/L)/sediment-%(particles/kg ww)]				
Fragment	91	87	96	97
	(0.15 ± 0.12)	(0.16 ± 0.10)	(110.9 ± 63.5)	(145.7 ± 112.10)
Fiber	9	8	3	3
	(0.02 ± 0.02)	(0.01 ± 0.02)	(3.7 ± 5.6)	(7.7 ± 13.7)
Film	ND ***	5	1	ND ***
		(0.01 ± 0.02)	(0.5 ± 2.0)	
Size [unit: seawater-%(particles/L)/sediment-%(particles/kg ww)]				
0.02–0.3 mm	87	85	92	93
	(0.15 ± 0.11)	(0.15 ± 0.10)	(105.1 ± 59.3)	(113.5 ± 82.3)
0.3–0.6 mm	6	10	5	4
	(0.01 ± 0.01)	(0.02 ± 0.02)	(5.9 ± 5.0)	(6.7 ± 9.2)
0.6–1.0 mm	4	2	1	1
	(0.01 ± 0.01)	(0.01 ± 0.01)	(1.6 ± 2.4)	(0.9 ± 2.1)
1.0–5.0 mm	3	3	2	2
	(0.00 ± 0.01)	(0.01 ± 0.01)	(2.4 ± 2.8)	(3.3± 8.2)

* Only the polymer types detected in this study (PE, PS, PP, PY, acrylic, alkyd, PET, PVC, and nylon) are presented. ** Only the microplastic shapes observed (fragment, fiber, sphere, sheet, and pellet) are included. *** not detected.

## Data Availability

The original contributions presented in this study are included in the article. Further inquiries can be directed to the corresponding author.
